# Upcycling Discarded Apples into Cider: Yeast and Nutrient Strategies Shaping Fermentation and Sensory Quality

**DOI:** 10.3390/foods15122053

**Published:** 2026-06-06

**Authors:** Catarina Marques-Gomes, Beatriz Cardeal, António Inês, Fernanda Cosme, Virgílio Falco, Alice Vilela

**Affiliations:** 1 Chemistry Research Centre-Vila Real (CQ-VR), Department of Agronomy, School of Agrarian and Veterinary Sciences, University of Trás-os-Montes and Alto Douro (UTAD), 5000-801 Vila Real, Portugal; avimoura@utad.pt; 2 Department of Agronomy, School of Agrarian and Veterinary Sciences, University of Trás-os-Montes and Alto Douro (UTAD), 5000-801 Vila Real, Portugal; beacardeal@gmail.com; 3 Chemistry Research Centre-Vila Real (CQ-VR), Department of Biology and Environment, School of Life Sciences and Environment, University of Trás-os-Montes and Alto Douro, 5001-801 Vila Real, Portugal; aines@utad.pt (A.I.); fcosme@utad.pt (F.C.); 4 Centre for the Research and Technology of Agro-Environmental and Biological Sciences (CITAB), University of Trás-os-Montes and Alto Douro (UTAD), 5000-801 Vila Real, Portugal; vfalco@utad.pt; 5 Institute for Innovation, Capacity Building and Sustainability of Agri-Food Production (Inov4Agro), University of Trás-os-Montes and Alto Douro (UTAD), 5000-801 Vila Real, Portugal; 6 LAQV-REQUIMTE—Laboratory for Green Chemistry (LAQV) of the Network of Chemistry and Technology (REQUIMTE), University of Porto, 4050-313 Porto, Portugal

**Keywords:** discarded apples, cider fermentation, food waste valorization, nutrient management, non-*Saccharomyces* yeasts, mixed fermentation, sensory quality

## Abstract

The increasing volume of discarded apples generated by commercial grading standards and postharvest losses represents both an environmental burden and an opportunity for sustainable valorization. Despite growing interest in circular economy strategies in the fruit-processing sector, a comprehensive review of the technological, microbiological, and nutritional factors influencing cider production from discarded apples remains limited. To address this gap, this review discusses key aspects of cider production from discarded apples, focusing on raw material characterization, nutrient management, yeast strategies, and fermentation technologies. The physicochemical and microbiological properties of discarded apples are examined, including soluble solids, acidity, phenolic composition, and microbial spoilage risks. Special attention is given to nutrient optimization, particularly yeast assimilable nitrogen (YAN), vitamins, and minerals, as deficiencies may cause sluggish fermentation and adversely affect volatile compound formation and stability. This review evaluates yeast selection, comparing Saccharomyces cerevisiae with non-*Saccharomyces* yeasts and mixed fermentations, highlighting their effects on chemical composition, aroma, and sensory quality. Innovative approaches such as yeast immobilization and repeated-batch fermentation are reviewed as tools to improve process performance. Key technical challenges, including variability in raw material quality, nutrient supplementation needs, contamination risks, and process scalability, are discussed alongside opportunities for valorization of cider pomace within a circular economy framework.

## 1. Introduction

Food loss and waste constitute a major global challenge, with far-reaching environmental, economic, and social implications. The management of agri-food waste remains particularly complex, as vast quantities of edible products are discarded each year due to aesthetic imperfections, overproduction, and inefficiencies along the supply chain [1]. Within the fruit sector, apples are among the most widely produced crops globally; however, a significant share of the harvest is rejected due to cosmetic defects, strict size classification standards, or postharvest deterioration. In Europe alone, the demand for visually “perfect” fruit and vegetables leads to the loss of around 30% of total production [2]. Despite their exclusion from fresh-market channels, these fruits often retain substantial nutritional and biochemical value, making them promising raw materials for developing value-added products. Consequently, the valorization of discarded apples has attracted growing attention as a sustainable strategy within circular economy frameworks, aiming to reduce food waste while generating new economic opportunities in the agri-food sector [3,4,5].

Cider production is one of the most promising pathways for upcycling surplus or downgraded apples. Fermentation processes can transform raw fruit material into a stable and commercially attractive beverage while preserving and enhancing its sensory and functional attributes. However, the physicochemical variability of discarded apples, including differences in sugar content, organic acids, phenolic composition, and nutrient availability, can significantly influence fermentation performance and the final quality of cider. These variations may affect yeast metabolism, fermentation kinetics, and the formation of key aroma compounds that define the beverage’s sensory profile [6,7,8].

The valorization of discarded apples, therefore, represents a distinct technological paradigm compared with traditional cider production. While conventional cidermaking relies on cultivar-specific apples with relatively stable chemical compositions, discarded apple streams typically consist of heterogeneous mixtures of dessert cultivars and post-harvest residues characterized by variable sugar, acid, and phenolic contents. Consequently, the central technological challenge is not the direct application of traditional cider-making practices, but rather the management of fermentation processes using chemically unpredictable substrates. Within this context, fermentation control strategies, including yeast selection, nutrient management, and process optimization, become essential tools for achieving fermentation stability and consistent product quality in beverages derived from upcycled apple resources.

Yeast selection and nutrient management are therefore critical factors in ensuring efficient fermentation and desirable product characteristics. Traditional cider fermentations are primarily driven by *Saccharomyces* species, particularly *Saccharomyces cerevisiae* and *Saccharomyces bayanus*, which are valued for their reliable fermentation, ethanol tolerance, and ability to produce desirable volatile compounds. More recently, non-*Saccharomyces* yeasts have attracted growing interest for their ability to modulate aromatic complexity, influence mouthfeel, and contribute to the development of differentiated cider styles. Nevertheless, these yeasts often exhibit lower fermentation efficiency and higher nutritional requirements, making appropriate nutrient management strategies essential for their successful application [9,10,11].

In apple must (apple juice), yeast-assimilable nitrogen (YAN), vitamins, and essential minerals such as magnesium and zinc are often present at low or highly variable concentrations, particularly when using discarded fruit or mixed apple batches [12,13]. Nutrient deficiencies can lead to sluggish or stuck fermentation, increased production of undesirable sulfur compounds, and reduced formation of desirable aroma molecules. Consequently, the implementation of targeted nutrient supplementation and optimized fermentation strategies is increasingly recognized as a key technological tool for improving fermentation performance and sensory outcomes in cider production [14].

Given the growing interest in sustainable food systems and resource valorization, a comprehensive understanding of the interactions among raw material composition, yeast metabolism, and nutrient management is essential to optimize cider production from discarded apples. This review synthesizes current knowledge on the use of discarded apples as substrates for producing fermented cider-type beverages, focusing on the interplay among raw material composition, yeast selection, and nutrient management during fermentation. Given the compositional heterogeneity of discarded apples, the review does not aim to replicate specific traditional cider styles but rather to evaluate technological strategies for producing stable, sensorially acceptable, and economically viable upcycled beverages. In this context, sensory quality is discussed in terms of fermentation consistency, aroma profile, sweetness–acidity balance, alcohol production, and consumer acceptability.

## 2. Discarded Apples as Raw Material for Cider Production

Discarded apples constitute a substantial proportion of total apple production and emerge at multiple rejection points throughout the supply chain. In commercial settings, fruit discarding is predominantly driven by appearance-based quality assessment, whereby visually suboptimal produce receives significantly lower consumer acceptance and purchase intention ratings, even when internal quality remains acceptable [15,16]. These findings highlight the dominance of visual perception over intrinsic compositional attributes in fresh-market decision-making.

Consumer behavior studies consistently demonstrate that both external defects, such as bruising, crushing, or splitting, and internal disorders, including browning and tissue breakdown, markedly reduce the probability of selection and increase the likelihood of discarding. For instance, the Belgian apple industry, worth 125–140 M euro, has experienced losses of 10–25%, corresponding to 10–25 M euro. Apple losses can be attributed to fungal diseases and vibrations occurring during transport [17]. Importantly, the severity of internal defects further amplifies rejection behavior. Collectively, this body of evidence indicates that discarding practices are largely governed by perceived quality deterioration rather than objective nutritional value or technological suitability [18].

In parallel with consumer-driven rejection, postharvest handling and transport conditions substantially contribute to fruit downgrading. Mechanical stresses, including vibration and impact during distribution, promote tissue disruption and bruising, thereby increasing susceptibility to fungal colonization and accelerating deterioration processes [17,19]. While such damage may compromise marketability in the fresh sector, it does not necessarily eliminate the biochemical attributes required for processing. This discrepancy underscores a critical mismatch between commercial grading criteria and the compositional parameters relevant for fermentation.

From a technological perspective, cosmetic and superficial structural defects do not inherently reflect the biochemical determinants of cider quality. Core attributes governing fermentation performance, namely soluble sugars, organic acids, phenolic compounds, and YAN, are largely independent of peel color, fruit size, or minor surface blemishes and are more dependent on the altitude of the apple production area, which affects apple quality, as studied by Zeng et al. [20] in Fugi apple ciders. Consequently, apples excluded from the fresh market may retain the intrinsic chemical composition necessary for successful cider production.

The physicochemical composition of apple juice is central to fermentation dynamics. Sugars determine potential alcohol yield; organic acids influence acid balance and microbial stability; and nitrogenous compounds and micronutrients regulate yeast growth, metabolic activity, and secondary metabolite formation [6]. Variability in these components, therefore, directly translates into differences in fermentation kinetics, aroma development, and final cider quality.

Compositional heterogeneity arises from multiple factors, including cultivar, geographical origin, altitude, ripening stage, and storage conditions. The study of hydroxycinnamic acids and flavonoids in apple juices and ciders was conducted by Laaksonenusing et al. [21] using liquid chromatography. Samples were derived from four distinct Estonian apple cultivars, utilizing unripe, ripe, and overripe apples, alongside six different commercial yeast strains, including *Saccharomyces cerevisiae*, *Saccharomyces bayanus*, and *Torulaspora delbrueckii*. A portion of the samples were further inoculated with malolactic bacteria, specifically *Oenococcus oeni*. The most significant distinction among the samples was the presence of phloretin in malolactic ciders, compared with conventional ciders and juices. Additionally, the apple cultivars exhibited considerable variation in phenolic content and composition, as noted by Calugar et al. [22]. It was observed that ciders and juices produced from unripe apples contained a higher concentration of phenolic compounds than those derived from ripe or overripe apples, although this effect varied based on the specific cultivar. The commercial yeast strains displayed variations in the release of free hydroxycinnamic acids (HCAs), specifically p-coumaric acid, during fermentation. In ciders fermented with *S. bayanus*, the concentration of p-coumaric acid was found to be higher compared to ciders fermented with *S. cerevisiae*, highlighting the significance of the fermentative yeast used [21].

Taken together, these findings demonstrate that raw material variability is not incidental but structurally embedded within apple production systems. When discarded apples originate from mixed sources, this heterogeneity becomes amplified, potentially influencing fermentation outcomes.

Beyond compositional parameters, microbial ecology adds another layer of complexity. The interaction between apple matrix composition and fermentative microorganisms plays a decisive role not only in the formation of aroma-active compounds but also in fermentation stability and safety [23]. Apple juice naturally harbors indigenous microbiota, including wild yeasts, lactic acid bacteria, acetic acid bacteria, and filamentous fungi, whose prevalence may increase in mechanically damaged or improperly stored fruit [6]. Tissue disruption associated with bruising enhances microbial access to nutrients, potentially elevating initial microbial loads prior to inoculation.

Such indigenous microorganisms may compete with inoculated *Saccharomyces cerevisiae*, thereby influencing nutrient availability, metabolite production, and overall process predictability [6,24]. While certain non-*Saccharomyces* yeasts may contribute positively to aromatic complexity, uncontrolled microbial proliferation can compromise fermentation stability, increase the risk of spoilage, and result in a cider with a unique volatile profile [22]. The colonization of fruit by fungi, particularly in cases of damage, raises significant safety concerns, notably the risk of mycotoxin contamination under inadequate storage conditions. As highlighted by Kumar et al. [23], the influence of host factors on the regulation of mycotoxin biosynthetic gene clusters underscores the complex interplay governing mycotoxin accumulation in ripening fruit. These considerations underscore the imperative of stringent sorting, sanitation, and meticulous management of fermentation processes when using downgraded fruit for cider production.

From a sustainability perspective, the valorization of discarded apples aligns with circular economy principles and resource recovery strategies. Apple-derived residues, such as apple peels, seeds, and pulp (solid residue of juice production) [7], have been widely recognized as reservoirs of bioactive and nutritional compounds, emphasizing the environmental burden associated with underutilized fruit biomass [25,26]. Apple pomace has been used in various ways, including extracting pectin, which serves as a gelling agent in jams and jellies. It is also added to cereal-based products like cookies and bread to enhance their dietary fiber content. Additionally, apple pomace can be fermented to create acetic acid-based products, such as vinegar [25]. These studies collectively support the broader rationale that downgraded apples retain substantial compositional value and may serve as suitable substrates for fermentation and other biotechnological applications.

Moreover, compositional variability among apple juices has been shown to extend to YAN, which is essential for yeast growth and fermentation metabolism. Quantitative analyses of apple juice fermentations reported YAN concentrations ranging from 9 mg N/L to 249 mg N/L, with most samples below 140 mg N/L, a threshold often considered necessary for robust fermentations in comparable systems [7]. Such variability in YAN, along with differences in sugar concentration, organic acids, and phenolic profiles associated with cultivar, maturity, and origin, can significantly influence yeast nutrient requirements, fermentation kinetics, and volatile compound formation. Consequently, when discarded apple streams consist of mixed maturity stages, cultivars, and storage histories, this heterogeneity may complicate nutrient management and process standardization.

To contextualize the technological implications of typical discard-related defects, it is necessary to consider the biochemical alterations associated with each defect category and their potential consequences for fermentation performance. Although systematic experimental comparisons remain limited, existing knowledge on apple physiology and fermentation science allows the establishment of plausible mechanistic links (Table 1).

## 3. Physicochemical Characterization of Discarded Apples

The physicochemical composition of apples strongly influences fermentation performance and the sensory quality of cider, particularly through parameters such as sugar concentration, acidity, phenolic compounds, and nitrogen availability [26]. Therefore, characterizing these attributes is essential when evaluating the suitability of discarded apples as substrates for cider production and other fermentation processes [27].

Apples rejected from the fresh market due to cosmetic defects or minor mechanical damage often retain fermentable sugars, organic acids, phenolic compounds, and micronutrients essential for yeast metabolism [26,28]. Consequently, their valorization through fermentation represents a promising strategy to reduce food waste while producing value-added beverages [5]. Physicochemical characterization, therefore, supports the optimization of fermentation strategies and facilitates the integration of discarded apples into sustainable cider production systems.

### 3.1. Physical Characteristics of Discarded Apples

Discarded apples frequently exhibit substantial variations in their physical characteristics due to differences in cultivar, maturity stage, and postharvest handling. Physical properties such as fruit size, weight, firmness, moisture content, and peel-to-pulp ratio directly influence both juice extraction efficiency and the composition of the apple juice used for fermentation [26]. Softer apples, often associated with advanced ripening or storage deterioration, may facilitate pressing and juice release but can also increase susceptibility to microbial contamination.

Apples typically contain approximately 80–85% water, which strongly influences juice yield and the concentration of soluble solids during pressing. Mechanical damage, bruising, or advanced ripening are among the most common causes of fruit rejection from the fresh market. Although bruised apples still contain fermentable sugars and other essential nutrients, tissue damage can accelerate enzymatic browning and microbial activity, potentially altering the juice’s chemical profile [29].

The distribution of compounds within the fruit also plays a significant role. Apple peel contains higher concentrations of phenolic compounds than pulp; therefore, apples with higher skin proportions may contribute to greater levels of tannins and antioxidants during juice extraction [30]. Consequently, variations in the proportion of apple skin may significantly affect the phenolic profile and sensory attributes of the final beverage. These compounds play an important role in cider quality by influencing color stability, mouthfeel, and oxidative resistance.

Recent studies further demonstrate that cider produced from different apple cultivars or apple from-products such as pomace exhibits significant differences in physicochemical and sensory characteristics, highlighting the importance of raw material selection [31]. While there is growing consensus on the potential of discarded apples as a sustainable substrate, findings vary depending on the processing technologies and control strategies employed [6,31].

Despite this potential of discarded apples, several technological limitations constrain their application. A major challenge is the heterogeneity of discarded raw materials, as discarded apples often consist of mixed cultivars with varying degrees of ripeness and quality. This variability complicates process standardization and can lead to inconsistent fermentation outcomes. Additionally, extracting fermentable sugars from by-products such as pomace is inherently less efficient and often requires enzymatic treatments, such as pectinases, to improve yield. Fermentation may also be affected by lower levels of yeast-assimilable nitrogen and the presence of inhibitory phenolic compounds, necessitating nutrient supplementation and careful process control.

From a practical perspective, using discarded apples in cider production involves balancing sustainability and product quality. While the valorization of waste streams supports circular economy principles and reduces raw material costs, it may also compromise sensory consistency and consumer acceptance. To address these challenges, producers often rely on blending strategies or technological interventions to achieve desired flavor profiles. Additionally, regulatory frameworks and market expectations for cider quality may limit how much lower-grade raw materials can be incorporated.

### 3.2. Chemical Characteristics of Discarded Apples

#### 3.2.1. Sugar Composition and Fermentable Substrates

Sugars are the primary substrates for yeast metabolism during alcoholic fermentation. The main sugars present in apples are fructose, glucose, and sucrose, with fructose generally being the most abundant [32]. The concentration of these sugars is commonly expressed as total soluble solids (°Brix), which directly correlate with the potential alcohol yield of cider.

Discarded apples often maintain sugar concentrations comparable to those of marketable fruit, particularly when rejection is based on visual rather than biochemical defects. However, in cases where apples have undergone degradation or prolonged storage, sugar levels may decline due to respiration and microbial activity, potentially reducing fermentation efficiency. The sugar profile is also strongly influenced by the stage of ripeness and storage conditions. During ripening, starch reserves are progressively hydrolyzed into simple sugars, increasing soluble solids content. For example, the sugar content in the juices of ‘Fuji Suprema’ and ‘Gala’ apples has been reported to increase by 26.8–36.7% from the unripe to the senescent stage; however, this increase is not uniform across individual sugars. Consequently, overripe apples may exhibit elevated total sugar levels, which can promote rapid fermentation. Nevertheless, such conditions may also require careful management of yeast nutrition to ensure balanced, complete fermentation [26].

#### 3.2.2. Organic Acids, pH, and Titratable Acidity

Organic acids are key determinants of cider flavor, microbial stability, and fermentation dynamics. Among them, malic acid is the predominant organic acid in apples. It is largely responsible for the characteristic tartness of apple juice and cider. The concentration of malic acid significantly influences both the pH and the titratable acidity of apple juice, thereby affecting yeast metabolism, fermentation kinetics, and the overall sensory balance of the fermented beverage [33].

In apples, malic acid is present at concentrations ranging from approximately 0.1 to 2.5 g per 100 g of juice, depending on cultivar, maturity stage, and growing conditions. Although malic acid is the dominant acid, apple juice contains several other organic acids in smaller quantities, including citric, succinic, citramalic, shikimic, glyceric, glyoxylic, isocitric, glycolic, lactic, and galacturonic acids. Additionally, keto acids such as oxaloacetic, pyruvic, and α-ketoglutaric acids may also be present, although they represent only a minor fraction of the total organic acid content. Citric acid is found at concentrations that are significantly lower than those of malic acid. Citric acid, in particular, is present at significantly lower concentrations than malic acid [34].

Titratable acidity, usually expressed as grams of malic acid per liter, represents the total acid concentration and plays a fundamental role in the sensory balance of cider, contributing to freshness, flavor complexity, and microbial stability. In discarded apples, the concentration of organic acids may vary considerably with storage duration and physiological maturity. During prolonged storage or advanced ripening, malic acid can be gradually metabolized through respiratory processes, leading to a reduction in acidity. These changes may alter the juice’s pH and acid balance, potentially affecting both fermentation performance and the final cider’s sensory profile.

While most studies consistently report malic acid as the dominant acid, discrepancies persist regarding its stability during processing. Some authors describe significant degradation during storage and fermentation, whereas others report relatively stable levels, depending on processing conditions and microbial activity [35]. These contrasting findings highlight the importance of carefully monitoring organic acid composition when assessing the suitability of discarded apples for cider production.

#### 3.2.3. Nitrogen Compounds and Yeast Nutrition

Nitrogen availability is a critical factor affecting yeast growth, fermentation kinetics, and the production of volatile aroma compounds during cider fermentation. In apple juice, nitrogen is primarily present as amino acids and ammonium ions, collectively referred to as Yeast Assimilable Nitrogen (YAN), which represents the fraction of nitrogen available for yeast metabolism. YAN includes ammonia/ammonium and α-amino nitrogen, and is essential for efficient, complete fermentation. Typically, YAN levels in apple juice range from 27 to 574 mg/L, and are often considered the primary limiting factor for yeast growth [36,37].

This limitation may be more pronounced in discarded apples, particularly those subjected to prolonged storage or degradation, where nutrient depletion may occur. Insufficient nitrogen availability can result in sluggish or stuck fermentations and promote the formation of undesirable compounds such as hydrogen sulfide. For this reason, cider fermentation often requires nutrient supplementation, particularly when low-nutrient substrates such as clarified apple juice or fruit by-products are used. Understanding the nitrogen composition of these raw materials is therefore essential for designing effective fermentation strategies, including appropriate yeast strain selection and nutrient management [32].

The variability and frequently limited concentration of YAN in apple juice have important practical implications for fermentation control. To mitigate the risks associated with nitrogen deficiency, cider producers commonly apply supplementation strategies, using either inorganic nitrogen sources, such as diammonium phosphate, or complex organic nutrients containing amino acids, peptides, vitamins, and micronutrients. The choice and timing of supplementation are critical, as they directly influence yeast metabolism and aroma development [8,38,39].

Organic nitrogen sources generally support more balanced yeast growth and enhance the production of desirable volatile compounds, including esters and higher alcohols, which contribute to the complexity of cider aroma. In contrast, excessive or poorly timed additions of inorganic nitrogen may alter metabolic fluxes, favoring rapid biomass formation over the synthesis of secondary metabolites, thereby reducing aromatic diversity. Consequently, targeted nitrogen management, based on initial YAN measurement and staged nutrient additions during early phases of fermentation, is increasingly recommended to optimize fermentation performance while preserving desirable sensory characteristics in cider production [8,38,39].

Although nutrient supplementation is widely applied to support yeast growth, prevent sluggish fermentation, and modulate the production of aroma compounds, it is important to recognize its limitations. Nutrient management primarily helps stabilize fermentation performance when using substrates with variable composition. However, it cannot recreate the intrinsic phenolic profile, tannin content, or compositional balance characteristic of traditional cider apple cultivars, which are largely determined by genotype and orchard conditions.

In the context of discarded apple valorization, technological interventions should therefore be understood mainly as tools for process stabilization and fermentation control, enabling consistent fermentation behavior despite the compositional variability inherent to heterogeneous apple streams.

#### 3.2.4. Phenolic and Bioactive Compounds

Polyphenols are important secondary metabolites in cider apple fruits and play a key role in several sensory attributes of cider, including color, bitterness, astringency, and colloidal stability [26,33]. Apples contain a wide range of phenolic compounds, including flavonoids, phenolic acids, and tannins, which contribute to the sensory and functional properties of cider [30]. These compounds are primarily concentrated in the apple peel and seeds, although they are also present in the pulp. During juice extraction and fermentation, phenolics may undergo oxidation or enzymatic transformations that alter their concentration and sensory impact. The phenolic profile of apples varies widely depending on cultivar, maturity stage, and processing conditions [40,41].

Discarded apples may still retain significant levels of phenolic compounds, particularly when rejection is due to external defects rather than internal deterioration. However, bruising and tissue damage may accelerate oxidative reactions, leading to enzymatic browning and potential degradation of some phenolic compounds [30].

## 4. Microbiological Aspects and Safety Challenges

### 4.1. Microbiological Aspects of Cider Production

Despite the growing worldwide production and consumption of fermented apple beverages, research on the composition and dynamics of the microbial population in cider remains relatively scarce compared with wine production. Cider production has distinct attributes across producing countries, with French ciders among the most notable and best-studied traditional examples. The cider microbiota comprises multiple microorganisms that affect both the quality and safety of the final product [42,43]. Amid this wide biodiversity, microbial pathogens and toxic by-products of microbial origin, including mycotoxins, biogenic amines, and ethyl carbamate, can significantly reduce the safety of consumed products [44]. Accordingly, comprehensive knowledge and proper management of the microbiological dynamics of cider production are critical to guarantee both product quality and consumer safety.

Cider production relies on many microorganisms, mainly yeasts and lactic acid bacteria (LAB). A systematic approach to biotechnological cider-making shows that yeast and bacterial cultures strongly affect both the sensory properties and the microbiological stability of this beverage. The most common yeasts used are *Saccharomyces cerevisiae* and *Saccharomyces bayanus*. *Saccharomyces bayanus* is valued for its quick fermentation (up to 7 days at 10–12 °C), low residual sugar levels (0.2–0.4%), and its fruity-floral flavor [45]. In addition to traditional *Saccharomyces* species, non-*Saccharomyces* yeasts such as *Metschnikowia pulcherrima* and *Torulaspora delbrueckii* [45], *Rhodotorula mucilaginosa*, *Debaryomyces hansenii*, *Zygosaccharomyces bailii*, and *Kluyveromyces marxianus* [46], *Pichia kudriavzevii* [47], *Pichia kluyveri*, and *Hanseniaspora vineae* [48] have attracted significant interest in cider production. These yeasts contribute distinct metabolic activities and sensory attributes to the final product, making them a strategy for improving cider flavor. Moreover, when co-fermented with *S. cerevisiae* and *Lactiplantibacillus plantarum*, these microorganisms increase the concentrations of complex esters, glycerol, and antioxidants, thereby improving the sensory qualities of cider [45,47]. Furthermore, lactic acid bacteria, particularly *Oenococcus oeni* and *Lactiplantibacillus plantarum*, enhance microbiological stability through acidification and the production of antimicrobial metabolites [49] and promote beneficial biological activities [50]. Also, according to the results of Călugăr et al. [51], yeasts and lactic acid bacteria in mixed fermentations provide a sustainable method, shorten fermentation duration, and allow successful application in the cider industry. Microorganisms associated with cider spoilage, such as *Zymomonas mobilis*, *Lactobacillus* sp., or *Brettanomyces*/*Dekkera* sp., can be detected early by monitoring microbial diversity through metagenomic approaches and physicochemical analyses. This strategy shows promise for improving cider fermentation performance [43].

### 4.2. Safety Challenges in Cider Production

A primary safety concern in cider production is the risk of contamination by harmful microorganisms and the formation of toxic metabolites. Spontaneous and traditional fermentation processes, which rely on native microflora rather than controlled starter cultures, pose specific health risks [44]. Unlike controlled fermentations with starter cultures, spontaneous fermentations lack the predictability and safety measures necessary to ensure beneficial microorganisms dominate over potential pathogens [44]. This issue is especially relevant in artisanal cider production, where traditional methods relying on spontaneous fermentation are becoming more common, thereby undermining safety controls [52]. The danger of pathogenic bacteria contaminating cider during production is a significant concern. Major foodborne pathogens that threaten fermented beverages include various bacterial, yeast, and mold species. Microbial contamination can occur through multiple routes, including contaminated water, improper handling, incorrect processing temperatures, and cross-contamination from equipment [53]. These contamination events can result in the presence of vegetative pathogens such as *Escherichia coli* and *Salmonella enterica* subsp. *enterica* serovar Enteritidis, and *Listeria monocytogenes*, all posing serious public health risks [54]. Although low pH in cider and similar beverages can suppress the growth of some pathogens, the risk remains, especially in products with incomplete fermentation or poor preservation, as demonstrated by a strain of *E. coli* O157:H7 in studies by Miller and Kaspar [55]. Therefore, chemical treatments such as decarbonate (DMDC) and sulfur dioxide (SO_2_) [56], along with physical methods like High Pressure Processing (HPP), have been recommended to eliminate various strains of *Escherichia coli* O157:H7 in apple ciders and other foods and beverages, as well as other foodborne microorganisms, including *Staphylococcus aureus*, *E. coli*, *Listeria monocytogenes*, and *Salmonella* [57].

Another safety concern in traditional cider production is the presence of mycotoxins, particularly patulin, in raw apples. Patulin, produced mainly by species of *Penicillium*, *Aspergillus*, and *Byssochlamys*, is a significant public health concern because it is frequently detected in commercial fruit juices and apple-derived products [58]. Consumption of contaminated apple juice or cider may lead to both acute and chronic health effects [59]. Among patulin-producing fungi, *Penicillium expansum* is considered the principal source of contamination in apples, especially in damaged, bruised, or decaying fruit tissues [60]. Another relevant species is *Paecilomyces variotii*, the anamorph of *Byssochlamys nivea*, which poses an additional challenge due to its heat resistance, persistence in fermentative environments, and tolerance of sanitation procedures [61].

Patulin contamination often develops before processing, particularly during storage, when fungal proliferation can lead to significant accumulation of the toxin directly in the fruit. During juice extraction and fermentation, patulin may partially transfer into apple juice and cider. Because the toxin is relatively stable under acidic conditions, it can persist throughout processing if contaminated fruit is used [62,63]. For this reason, regulatory authorities have established strict limits for patulin in apple products. The European Union sets a maximum level of 50 μg kg^−1^ for fruit juices and cider, with lower limits for products intended for infants. Similar thresholds are adopted internationally [64,65].

To minimize contamination risks, mitigation strategies primarily focus on preventing contamination of raw materials and controlling processing conditions. Effective pre- and post-harvest practices include proper sanitation, gentle fruit handling to avoid tissue damage, rapid removal of compromised fruit, and strict temperature control during storage [58]. In cider production, additional measures such as rigorous sorting of mold-infected apples, washing and trimming damaged tissues, and rapid processing after harvest are essential to limit fungal growth and toxin formation. Technological interventions, including clarification, filtration, fermentation, and adsorption treatments, may further reduce patulin concentrations, although their effectiveness depends on the initial contamination level and juice composition [62,63]. Consequently, strict raw material selection combined with good manufacturing practices remains the most effective strategy to ensure that patulin levels in cider products remain below regulatory limits.

Biogenic amines (BAs) are another food safety concern in cider production. They may accumulate during fermentation and trigger physiological and toxic reactions [62]. The main BAs produced by Lactic Acid Bacteria via amino acid decarboxylation are histamine (from histidine), tyramine (from tyrosine), and putrescine (from ornithine or agmatine) [63,64,65,66,67,68,69,70]. Consuming foods and beverages high in biogenic amines, especially histamine, poses health risks, including allergic-type responses and gastrointestinal issues [62]. The formation of biogenic amines depends on raw material composition, the microorganisms present during fermentation, and processing and preservation methods [62]. Biogenic amines are heat-stable and not eliminated by heating. However, their levels can be minimized by controlled fermentation with starter cultures [62] that have low decarboxylating activity and/or by good manufacturing practices, thereby hindering the conditions favorable to biogenic amine production [71].

## 5. Nutrient Management in Cider Fermentation

Efficient cider fermentation depends on the yeast’s ability to adapt metabolically to the nutritional composition of apple juice, which directly influences growth, fermentation kinetics, and metabolite formation [6]. Beyond fermentable sugars, *Saccharomyces cerevisiae* requires assimilable nitrogen, amino acids, vitamins (notably B-complex vitamins), and minerals to sustain biomass formation, enzymatic activity, and secondary metabolite production [6,72,73,74]. Reviews of apple juice fermentation systems emphasize that physicochemical composition strongly influences yeast metabolism, fermentation kinetics, and the formation of aroma-active compounds [6].

Nitrogen availability constitutes the primary metabolic regulator of yeast proliferation and fermentation kinetics in cider systems. In contrast to grape must, which typically exhibits YAN concentrations within ranges considered adequate for robust fermentations, apple juice is frequently characterized by lower, highly variable YAN levels, often falling below 140 mg N/L [8,72]. Reported YAN concentrations in apple juice range from as low as 9 mg N/L to 249 mg N/L, with substantial inter-sample variability [72]. This inherent compositional variability positions nutrient management as a central technological determinant in cider production rather than a secondary corrective measure.

In cider systems, YAN, commonly defined as the sum of ammonium (NH_4_^+^) and primary amino nitrogen (PAN), directly influences biomass formation and fermentation dynamics, including growth rate and metabolic homeostasis [7]. This documented variability reflects differences in cultivar, agronomic conditions, maturity stage, and postharvest handling [73].

Low YAN levels can compromise fermentation efficiency by limiting yeast biomass formation and reducing sugar uptake rates, thereby increasing the risk of sluggish or incomplete fermentation. In addition to kinetic effects, nitrogen limitation alters sulfur metabolism in *S. cerevisiae*. Hydrogen sulfide (H_2_S) is produced as an intermediate of the sulfate reduction sequence (SRS), in which sulfate is reduced to sulfide and subsequently incorporated into sulfur-containing amino acids such as cysteine and methionine. Under nitrogen-limited conditions, the incorporation of sulfide into amino acids may become constrained, leading to intracellular sulfide accumulation and its release as H_2_S. In cider fermentation, nitrogen availability has been shown to significantly influence H_2_S formation, linking nutrient status to sulfur-related off-aromas. Importantly, the relationship between YAN and H_2_S production is not strictly linear, reflecting complex regulatory interactions between nitrogen and sulfur metabolism and strain-dependent responses [74].

While nitrogen is the most extensively studied nutrient in cider fermentation, micronutrients, including vitamins and minerals, also play essential roles. Thiamine (vitamin B_1_) is a key cofactor in central carbon metabolism and has been identified as critical for yeast performance during alcoholic fermentation [10]. Thiamine limitations can alter metabolic fluxes and influence fermentation outcomes.

Recent metabolomic and biochemical studies demonstrate that vitamin availability modulates volatile compound production by directly influencing central carbon metabolism and redox balance in *S. cerevisiae* [75,76]. Thiamine, in its active form thiamine pyrophosphate (TPP), functions as an essential cofactor for enzymes such as pyruvate decarboxylase (PDC), which catalyzes the decarboxylation of pyruvate to acetaldehyde [73,76]. This reaction represents a key metabolic node linking glycolysis to ethanol and higher-alcohol synthesis. Thiamine limitation can reduce PDC activity, leading to altered acetaldehyde accumulation, impaired NAD^+^ regeneration, and disruption of redox balance [73]. Such metabolic perturbations may alter the distribution of carbon flux and, consequently, influence the formation of higher alcohols and acetate esters derived from pyruvate metabolism [75,76].

Biotin also plays an essential role in yeast metabolism, participating in carboxylation reactions and lipid biosynthesis pathways that are critical for membrane composition and cellular stress tolerance [75]. Because membrane integrity affects nutrient transport efficiency and ethanol tolerance, variability in biotin availability may indirectly modulate fermentation robustness and aroma development.

In addition to vitamins, mineral cofactors such as magnesium (Mg^2+^) and zinc (Zn^2+^) are fundamental for enzymatic stability and metabolic regulation. Magnesium stabilizes ATP and supports the activity of multiple glycolytic enzymes and kinases, whereas zinc serves as a structural and catalytic cofactor of alcohol dehydrogenase and transcriptional regulators involved in metabolic control [11]. Deficiencies in these micronutrients can compromise enzymatic efficiency, impair sugar metabolism, and affect ethanol production and volatile compound synthesis.

Variability in micronutrient availability, particularly in heterogeneous apple streams derived from different cultivars, maturity stages, or storage conditions, may therefore modulate yeast resilience, fermentation stability, and sensory outcomes. Although much of the mechanistic evidence originates from wine systems, the conserved physiology of *S. cerevisiae* supports the direct applicability of these biochemical principles to cider fermentation [11,73,76].

Nutrient deficiencies may manifest as prolonged lag phases, reduced fermentation rates, and an increased risk of sluggish or incomplete fermentations. In cider systems, insufficient YAN limits biomass formation and sugar uptake capacity, thereby compromising fermentation kinetics and process predictability [72,74]. Beyond kinetic effects, nitrogen limitation can contribute to sulfur-derived off-aromas and increased batch-to-batch sensory variability [74]. From a technological standpoint, inadequate nutrient availability therefore represents not only a metabolic constraint but also a critical determinant of fermentation consistency and product quality.

Given the variability and frequent insufficiency of YAN in apple must, supplementation strategies are widely investigated to stabilize fermentation and modulate aroma formation. Comparative studies evaluating inorganic nitrogen sources (e.g., diammonium phosphate, DAP) and organic nitrogen sources (e.g., amino acid mixtures) demonstrate that both can improve fermentation performance but may differentially affect the volatile composition [12]. Experimental work in nitrogen-poor apple and pear mashes further confirms that nitrogen supplementation accelerates fermentation, increases yeast biomass, and modifies aroma profiles [13]. These findings indicate that nitrogen source selection influences not only fermentation kinetics but also sensory outcomes.

Emerging approaches also explore circular nutrient management strategies, including the reutilization of processed yeast lees as nutrient supplements in subsequent fermentations [3,4,77]. Lees, the nutrient-rich sediment resulting from alcoholic fermentation, can be autolyzed or lysed to release amino acids, peptides, vitamins, and trace elements, which are suitable for reuse as fermentation nutrients [4]. Experimental studies have demonstrated that adding processed lees preparations can improve fermentation kinetics and modulate volatile compound production in a dose-dependent manner, illustrating the feasibility of nutrient recycling from fermentation by-products [3,77]. Such strategies are particularly relevant in cider production from discarded apple streams, where nutrient composition is inherently variable and frequently limiting.

Importantly, nutrient supplementation should not be applied uniformly. Given the non-linear relationship between YAN and metabolite production [74], effective management requires initial nutrient assessment, appropriate source selection, and controlled timing of additions to balance fermentation robustness with sensory quality.

When cider production relies on discarded or heterogeneous apple streams, nutrient management becomes even more critical. Mixed maturity stages, cultivars, and storage histories can generate substantial variability in nitrogen availability and overall nutrient composition. Considering the documented range of YAN concentrations in apple juice [72], uncorrected variability may translate into inconsistent fermentation performance and aroma outcomes.

Therefore, nutrient management in cider should be conceptualized as a dynamic control lever integrating compositional assessment, metabolic understanding, and targeted supplementation strategies. Rather than serving solely as a corrective measure, it represents a proactive framework for ensuring robust fermentation, sensory consistency, and technological adaptability amid increasingly variable raw material streams.

**Figure 1:**
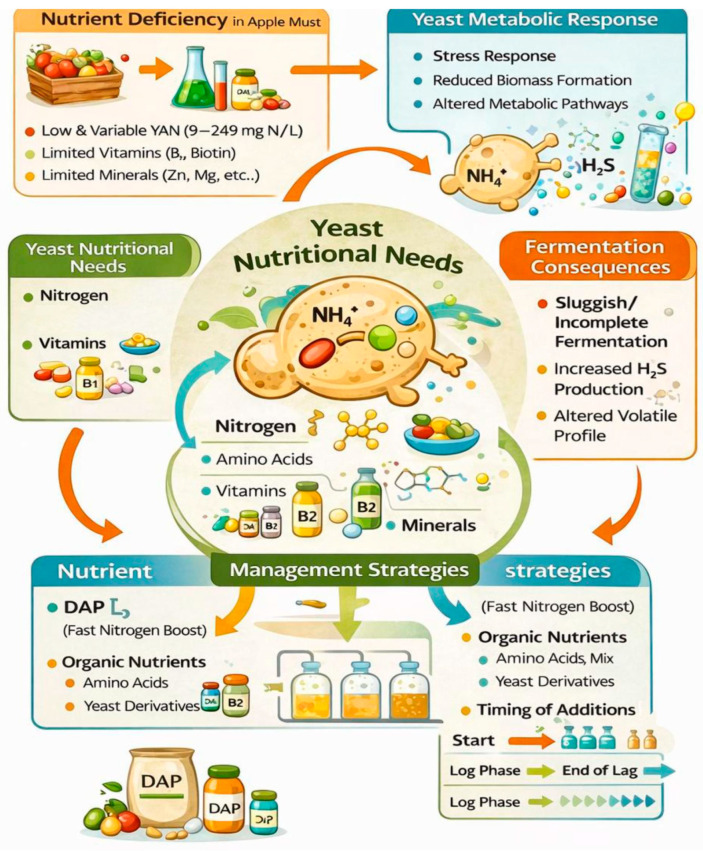
Nutrient management framework in cider fermentation. Adapted from [6,72,74].

The key elements of nutrient management in cider fermentation, along with their interactions with yeast metabolism and fermentation outcomes, are summarized in Figure 1.

## 6. Yeast Strategies for Cider Production

Yeast selection and management are central to cider production, as they directly influence fermentation efficiency, flavour development, aroma complexity, and overall product stability. Unlike wine or beer, cider fermentation presents unique challenges due to the naturally low nitrogen content, variable sugar composition, and acidic profile of apple juice. As a result, targeted yeast strategies are required to ensure consistent, high-quality cider.

### 6.1. Core Choice: Saccharomyces vs. Non-Saccharomyces

Yeast strategies are a critical determinant of fermentation performance and sensory quality in cider production, as yeast metabolism governs sugar conversion, aroma formation, alcohol yield, and chemical stability. Apple juice presents specific constraints for fermentation, including low assimilable nitrogen, high acidity, and variable sugar profiles, necessitating careful yeast strain selection and tailored fermentation management.

Among *Saccharomyces* species, *Saccharomyces bayanus* is widely recognized for its robustness and technological reliability. Strains of *S. bayanus* are characterized by rapid and complete fermentations, low residual sugar levels, and clean fruity–floral sensory profiles. Cryotolerant hybrids are particularly advantageous for low-temperature fermentations (10–13 °C), making them well-suited for sparkling cider production and aroma retention [45,78,79]. In contrast, selected *Saccharomyces cerevisiae* strains are often chosen for their capacity to achieve high ethanol yields and enhanced ester production, contributing to more intense aromatic profiles and increased antioxidant activity. Commercial strains such as *La Raffinée* and WFC-SC-071/072 exemplify these attributes and are commonly applied in modern cider styles [80,81]. Table 2 presents some yeast strategies that can be used to achieve certain cider-style goals.

In recent years, non-*Saccharomyces* yeasts—including *Hanseniaspora*, *Pichia*, *Metschnikowia*, *Torulaspora*, *Wickerhamomyces*, and *Schizosaccharomyces*—have gained attention for their ability to modulate cider complexity. When used alone, these yeasts often exhibit slow or incomplete fermentations and limited ethanol tolerance; however, they exert strong influences on ester formation, higher alcohols, glycerol production, antioxidant capacity, and, in some cases, biological deacidification [46,47,82,83,84,85]. Importantly, targeted nutrient supplementation, particularly with magnesium, zinc, and nitrogen, can improve fermentation completeness in certain non-*Saccharomyces* species, thereby expanding their practical application in cider production [82].

**Table 2:** Matching yeast strategies to cider style goals.

Goal/Style	Yeast Strategy	Typical Effects	Ref.
Reliable dry cider	Pure *S. cerevisiae*/*bayanus*	Complete sugars, predictable aroma, simpler profile	[43,80,81]
Complex fruity/floral	Mixed *S. cerevisiae* + *Hanseniaspora*/*Pichia*/*Metschnikowia*/*Wickerhamomyces*	More esters, phenethyl acetate, fruity/floral notes, higher glycerol	[46,47,84,85,86]
Lower acidity/softer mouthfeel	*Schizosaccharomyces* spp., *Pichia kudriavzevii*, *Metschnikowia koreensis*, LAB co-ferment	Malic to lactic acid conversion, higher lactic/succinic, reduced sourness	[47,51,85,86,87]
Low-temp/sparkling	Cryotolerant *Saccharomyces* (Su, Se) or Sc × Su/Se hybrids; encapsulated yeast	Robust low-T fermentation, distinct aromatic profiles, and easier riddling	[78,79,88]

### 6.2. Inoculation Patterns and Co-Fermentation

Simultaneous or sequential co-inoculation strategies combining *S. cerevisiae* with non-*Saccharomyces* yeasts have consistently been shown to increase the complexity of cider aroma compared with monoculture fermentations. These mixed fermentations promote a broader and more balanced volatile profile, particularly by increasing the production of fruity and floral esters, higher alcohols, and secondary metabolites that contribute to sensory depth [46,47,48,51,85,86,89]. Sequential inoculation is especially effective at enabling non-*Saccharomyces* yeasts to express their metabolic potential during early fermentation while maintaining complete fermentation through subsequent *S. cerevisiae* dominance.

The success of co-inoculation is strongly dependent on inoculation ratios and timing. Balanced inoculation ratios, such as 1:1 (*S. cerevisiae*: *Wickerhamomyces* or *Pichia*), have been reported to maximize ester synthesis without compromising fermentation kinetics. In sequential approaches, delaying *S. cerevisiae* inoculation by approximately 31 h following *Hanseniaspora* introduction significantly increases the formation of key aroma compounds, including 2-phenylethyl acetate, which is associated with rose-like and honeyed notes [48,86,89]. More complex consortia, such as triple cultures involving *S. cerevisiae*, *Pichia kudriavzevii*, and *Lactiplantibacillus plantarum*, further enhance ester concentration, antioxidant capacity, and overall sensory quality, while simultaneously moderating ethanol content and perceived acidity [47]. In addition, mixed yeast–lactic acid bacteria fermentations can shorten fermentation duration and increase lactic acid formation, resulting in softer acidity and more complex flavor profiles [45,47,51].

#### Spontaneous vs. Inoculated and Special Cases

Spontaneous cider fermentations typically begin with diverse populations of non-*Saccharomyces* yeasts and lactic acid bacteria, then gradually shift toward dominance by *Saccharomyces*. While this approach introduces higher variability and microbial risk, it often yields distinctive sensory profiles and strong regional identity [87,90]. Conversely, inoculated fermentations using selected *S. cerevisiae* strains suppress much of the native microbiota, reduce lag phase, and improve process reproducibility, though sometimes at the expense of aromatic complexity [83,87]. For non-alcoholic or low-alcohol cider styles, using specific yeast strains, such as lager yeasts like Saflager S-23, is an effective strategy to limit ethanol production while preserving characteristic apple aroma and freshness [91].

## 7. Fermentation Technologies and Process Optimization

For cider production, apples are sorted and washed to remove rotten fruit and orchard debris prior to milling and pressing to extract the juice. Under optimal conditions, juice yields of 70–80% (*w*/*w*) can be achieved, but yields may drop below 65% when apples have been stored beforehand, largely due to moisture loss during storage [92]. In response to rising cider production costs, there has been a need for more efficient juice extraction methods.

A range of pre-fermentation treatments can be applied to produce the highest-quality juice. For example, keeving, also called défécation, that often involves adding calcium chloride and pectin methylesterase (PME) to the juice, promotes protein denaturation and precipitation, along with partial pectin breakdown, helping to clarify the juice [33,93]. Other possible treatments include flash pasteurization and the addition of sulfur dioxide. More recently, pulsed electric field treatment, microwave extraction, ultrasound treatment, high-pressure processing, and ultraviolet treatment have also been used.

In conventional batch fermentation, yeast cells are freely suspended in a tank containing a fixed volume of unfermented apple juice. That full volume (the “batch”) is allowed to ferment, then drained from the vessel. Compared with freely suspended cultures, immobilized cell systems can improve fermentation performance by enabling continuous operation. In continuous fermentation, fresh juice is fed into the tank continuously while an equal flow of fermented product is removed. When yeast is immobilized on suitable supports, very high cell densities can be maintained in the fermentation tank, and together with high flow rates, this allows much shorter residence times.

### 7.1. Innovative Pre-Fermentative Treatments

Pulsed electric field (PEF) is a non-thermal technique that induces increased cell wall permeability in biological tissues, such as apple tissue [94,95]. PEF treatment of apple mash is an attractive approach for juice extraction, since it can enhance yield, potentially increase polyphenolic content, and improve clarity [96,97]. However, a rise in the nutritive value of the juices as a result of improved disintegration of the plant cells from the PEF-treated apple mash was not observed [98].

The effect of PEF on the inactivation of naturally occurring microorganisms in apple juice was investigated in a continuous-flow system. The microbial count decreased with an increase in applied pulses (17.6–58.7 total) and treatment temperature (45–50 °C), and a decrease in flow rate (3–10 L/h) [99]. The feasibility of inhibiting polyphenol oxidase in apples using PEF treatment was also evaluated. PEF treatment significantly lowered polyphenoloxidase activity in apple enzyme extracts. The temperature of the samples never exceeded 15 °C during pulsed electric field processing treatments. Polyphenoloxidase activity in apple extract was reduced to 3.15% of the initial value at 24.6 kV/cm over a total treatment time of 6 ms [100].

Microwave-assisted heating is a technique widely used to accelerate the release of polyphenolic compounds from the cell walls of plant tissues. For food applications, the approved and most used microwave frequencies are 2450 ± 50 and 915 ± 25 MHz [101]. This technique rapidly reaches high temperatures by selectively heating water molecules under microwave irradiation. To evaluate the effects of microwave heating of apple mash on juice yield and quality, apple mashes were heated to bulk temperatures of 40 °C, 50 °C, 60 °C, and 70 °C in a 2450 MHz microwave oven at 1500 W [102]. Juice yield increased when the mash was heated before pressing. While cider produced from heated mashes had comparable pH, titratable acidity, and sensory characteristics to cider produced from room-temperature mashes, the soluble solids, total phenolic, and flavonoid content of the juice increased with increasing mash temperature. The results of this study also showed that heating the apple mash to 60 °C is the optimum temperature for improving juice quality and yield.

Gentry and Roberts [103] designed a continuous flow microwave pasteurization system for apple juice. Process lethality was verified by inoculating *Escherichia coli* into apple juice, where microwave pasteurization achieved a 5-log10 reduction.

As an alternative to juice pasteurization, ultrasound, defined as sound waves with frequencies greater than 20 kHz, is recognized as a potential non-thermal technique for inactivating microorganisms in fruit juices [104,105]. Ultrasound treatment of apple juice, with or without mild heat (57 °C), was effective in reducing *Escherichia coli* levels [106]. However, the US Food and Drug Administration evaluated ultrasound for potential food-industry use and concluded that, on its own, it does not reliably inactivate many bacterial species and therefore is not suitable as a standalone preservation method [105]. In a study on the efficacy of Dynashock wave power ultrasound for the microbial inactivation of cloudy apple juice, within a 30 min exposure time to ultrasound, apple juice treatment did not result in the recommended 5-log10 reduction as stipulated in the Federal Juice HACCP, but co-treatment with UV-C irradiation significantly enhanced microbial inactivation [107]. Moreover, ultrasound pasteurization at 65 °C for 10 min using a 750 W probe sonicator resulted in a significant reduction in enzyme activities and complete inactivation of microbes in fresh pear juice [108]. Sonication, in combination with heat (55 °C and 59 °C) and low pressure (400 kPa), also accelerated the inactivation of *Escherichia coli* suspended in apple cider [109]. These results suggest that ultrasound has synergistic effects when combined with other food preservation methods, particularly that low-temperature pasteurization combined with ultrasound can be successfully employed for the commercial processing of fruit juices, improving quality.

High-pressure processing (HPP) applies elevated pressure, sometimes with added heat, to inactivate microorganisms or modify product characteristics. It is also known as high hydrostatic pressure (HHP) or ultra high-pressure (UHP) processing. HPP is a commercially viable alternative to thermal treatment by allowing food to be pasteurized at or near room temperature. High-pressure treatments can inactivate most pathogenic and spoilage vegetative microorganisms at about 200–600 MPa, using relatively mild processing temperatures [110]. In a study conducted to evaluate the combined effect of HPP and dimethyl dicarbonate (DMDC) to inactivate foodborne pathogens in apple juice, the pressure (ranging from 100 to 600 MPa), dwell time (from 26 to 194 s), and DMDC (from 116 to 250 mg/L) were tested [111]. Within the studied range, dwell time did not significantly affect pathogen reduction. Across all HPP and DMDC levels in this study, the combined use of HPP and DMDC achieved a >5-log10 reduction in *Escherichia coli* and *Listeria monocytogenes*.

HPP treatment can also inactivate enzyme activity in juices. A study by Marszalek et al. [112] showed that HPP (200–600 MPa/5–45 °C/1–15 min) decreased the activity of polyphenol oxidases (PPO) and peroxidases (POD) in cloudy apple juice, without significant changes in physicochemical parameters like pH, total soluble solids, sugars, and vitamin C. All HPP treatments in the study inactivated PPO and POD, and the highest pressure studied (600 MPa) caused almost complete inactivation of PPO and 58% inactivation of POD at 25 °C and 5 min. In contrast, in a study of the effectiveness of HPP to stabilize cloudy apple juices, HPP treatment up to 650 MPa at 25 °C failed to inhibit the activity of pectin methylesterase (PME) in a cloudy apple juice [113]. One possible explanation is that, in cloudy juices, PME is bound to suspended particles, which likely enhances its stability under pressure.

UV-C light in the 200–280 nm range has long been known to cause both direct and indirect damage to living cells, so it can be used for disinfection as an alternative to pasteurization [114]. Consequently, the U.S. FDA has approved UV-C irradiation for treating juice products to reduce levels of pathogens and other microorganisms [115]. UV-C treatment (254 nm) reduced about 99% of bacterial counts and 90% of yeast and mold counts in apple juice, meeting the requirements for commercial sterilization, without significantly affecting soluble solids, total sugars, or phenolics in the juice [116]. Also, UV-C radiation at 254 nm applied to apple juice drastically decreased the number of *Alicyclobacillus acidoterrestris* spores after 8 min and proved more effective than a 95 °C thermal treatment [117].

UV-C irradiation may also degrade the toxic mycotoxin patulin in apple juice, but high turbidity levels can reduce degradation rates, making this treatment impractical in unfiltered apple juice. Nevertheless, UV-C treatment (254 nm) reduced patulin concentrations in clarified apple juice, although the required doses were higher than those needed to achieve a 5 log10 reduction in pathogens [118,119]. Furthermore, UV-C radiation can affect some enzyme activities that are important in apple juice. For example, applying UV-C irradiation to freshly squeezed apple juice effectively inactivated polyphenol oxidase (PPO) after 100 min of exposure, and peroxidase (POD) after just 15 min [120].

### 7.2. Immobilized Cell Fermentation and Repeated-Batch Fermentation

Cell immobilization is the process of physically confining or localizing whole cells within a specific region of space while retaining the desired biological activity. Immobilization is analogous to the natural process by which cells adhere to and grow on surfaces within natural structures [121]. The immobilization support allows substrates, products, inhibitors, and other compounds to diffuse in and out, while keeping the active cell biomass separated from the surrounding bulk liquid phase. Over the past decades, a variety of techniques for immobilizing cells have been developed, and cell immobilization has proven effective in enhancing the performance of numerous fermentation processes [122,123]. Compared with freely suspended cultures, immobilized cell systems can improve fermentation performance by making it easier to separate the biomass from the liquid and facilitating product recovery. Furthermore, immobilized-cell fermentation enables faster fermentation by reducing the non-productive growth phase, increasing cell density, substrate concentration, cell productivity, and product yield. Immobilized-cell fermentation also shows improved cell resistance to inhibitory substrates or products and can be adapted to batch, fed-batch, or continuous process operation [124]. Hence, this technique has significant potential for the efficient production of fermented beverages such as cider.

One of the earliest demonstrations of the use of immobilized yeast for continuous apple juice fermentation, providing the engineering precedent for later cider bioreactors, was the continuous conversion of the sugar content of an apple juice into ethanol by *S. cerevisiae* entrapped in Ca-alginate gel [125]. The average values characterizing the process were: fermentation efficiency, 84.7 ± 4.2%; ethanol concentration in the mash, 38.9 ± 1.9 g/L; and volumetric productivity, 6.3 ± 0.5 g/L/h.

The use of immobilized-cell systems allows co-immobilization of different microorganisms within the same porous matrix, enabling both fermentation stages to be carried out in a single integrated process. An early explicit focus on flavor control via co-immobilized consortia, anticipating modern co-culture design (alcoholic + malolactic integration), was the immobilization of both *S. cerevisiae* and *Lactobacillus plantarum* in a sponge-like material for carrying out fermentation and partial maturation of alcoholic cider [126]. Nedovic and coworkers [127] used an integrated biocatalytic system consisting of co-immobilized *S. bayanus* and *Leuconostoc oenos* in a Ca-alginate matrix within a continuous packed-bed bioreactor to perform simultaneous alcoholic and malolactic fermentation of apple juice. The continuous process enabled much faster fermentation than the traditional batch process. Furthermore, by adjusting the flow rate of feeding substrate through the bioreactor, i.e., its residence time, it was possible to achieve different partial sugar conversions and obtain either “soft” or “dry” ciders. At a residence time of only 2 h, the consumption of sugar and malic acid was 65% and 70%, respectively; at 8 h, almost complete attenuation of both was achieved.

Immobilization techniques have recently been developed using less conventional immobilization materials. In a study aimed to valorize unqualified, unsold, and too mature melon, immobilized *S. cerevisiae* cells on melon rind peel waste, used as inoculum for repeated-batch fermentation, produced a fermented product with an alcohol concentration of 5.0–5.5%, with a fermentation cycle time of 24 h at 30 °C [128]. Scanning electron microscopy (SEM) images revealed the dense adhesion of yeast cells in the porous structure of the melon rind peel.

Immobilized cell systems offer clear technological and process advantages. By protecting cells from physicochemical stresses (such as pH and temperature shifts, heavy metals, or solvents), immobilization often extends cellular activity and stability [123]. It also enables much higher cell densities, which typically translates into greater productivity, improved substrate uptake, and higher yields, while increasing tolerance to high substrate loads and reducing the impact of product inhibition. High cell densities and enhanced fermentation activity also reduce the risk of microbial contamination [123]. From an operational and cost perspective, continuous immobilized-cell processing can substantially reduce fermentation time and “down-time,” while also simplifying parts of downstream operations (for example, less frequent filling, cleaning, and standby). At the same time, the key advantage of sustained continuous production can become a limitation: such systems are often less flexible than batch fermentations, and when disruptions occur, long restart times may offset gains achieved during stable operation [127]. Overall, immobilized cell technology is a powerful route to high-rate, high-yield, and potentially continuous bioprocessing if the requirements for process control, operational robustness, and flexibility are carefully balanced.

## 8. Chemical and Sensory Quality of Cider from Discarded Apples

The chemical and sensory quality of cider is determined by the intrinsic composition of the apples used (sugars, acids, phenolics, aroma precursors), together with the biochemical transformations occurring during fermentation and maturation [31]. When discarded apples are used, variability in ripeness, bruising, storage damage, microbial load, and oxidation state may strongly affect juice composition and, consequently, cider quality [129,130,131]. Nevertheless, discarded apples often retain fermentable sugars and relevant phytochemicals, making them suitable for cider production when appropriate control measures are applied [132]. In addition, apple pomace and other by-products from cider processing may also be incorporated or valorized as ingredients, influencing cider composition and sensory traits [133,134,135].

The use of discarded apples may result in ciders with distinct sensory profiles compared to premium dessert apple ciders [129]. Higher phenolic extraction and oxidative development may yield more robust, structured beverages that more closely resemble traditional tannic cider styles [136,137]. Additionally, the possibility of incorporating pomace-derived compounds, either directly or indirectly via ingredient recovery, underscores the potential of cider-making as a platform for circular-economy innovation [135].

Apple by-products have been widely recognized as valuable resources for developing added-value foods and beverages, not only because of their phenolic content but also for their fiber, pectin, and fermentable substrates [138]. While some microbial valorization pathways, such as citric acid production from pomace using *Yarrowia lipolytica*, are not directly related to cider sensory quality, they confirm that apple pomace is a metabolically rich substrate and highlight its industrial relevance [139].

Recent evidence supports that cider made from discarded apples can achieve acceptable or even improved sensory outcomes when chemical balance is optimized [129,132]. Key quality markers include volatile ester composition, acidity equilibrium, and phenolic structure, which together define aroma intensity, freshness, mouthfeel, and visual appeal [31,130]. Therefore, discarded apples should not be viewed as inferior substrates but rather as raw materials that require targeted quality control to ensure consistent chemical and sensory performance in cider.

### 8.1. Physicochemical Drivers of Cider Quality from Discarded Apples

Cider quality is largely shaped by the balance between soluble solids, acidity, and phenolic composition, as these components influence fermentation kinetics and sensory attributes such as color, bitterness, and mouthfeel [20]. Enzymatic browning in apples results from polyphenol oxidase (PPO)–mediated oxidation of phenolic substrates, which is activated by tissue disruption due to mechanical damage and prolonged storage [140]. This enzymatic activity accelerates the conversion of monomeric phenolics into polymerized brown pigments, leading to darker juice color and modified phenolic profiles prior to fermentation. Studies on apple storage have shown that extended postharvest periods increase PPO activity and membrane damage, thereby facilitating interactions between oxidoreductases and phenolic compounds, thus enhancing browning reactions and depleting antioxidant capacity [140,141]. Variations in the degree of polymerization and concentration of apple procyanidins can significantly influence cider astringency, with higher-molecular-weight polymers contributing to enhanced drying sensations and altered mouthfeel [142].

In cider, phenolic compounds exert a dual technological and sensory role: they contribute to antioxidant capacity and color stability through their redox properties, while also significantly modulating sensory perception, particularly bitterness and astringency, depending on their concentration and degree of polymerization [142,143]. Apple pomace, which remains a rich, sustainable source of bioactive compounds, including polyphenols (60–118 mg GAE/100 g dw), flavonoids (2153–3734 mg/kg dw), and dietary fibers [144]. Green extraction studies confirm that apple pomace from cider processing retains high concentrations of phenolic acids (e.g., chlorogenic acid), flavonoids, and dihydrochalcones such as phloridzin, reinforcing its relevance as a reservoir of compounds that can influence cider quality if reintroduced into the cider-making chain or used as an ingredient source for functional enrichment strategies [132,145].

### 8.2. Aroma and Volatile Composition

The aroma of cider is mainly driven by volatile compounds, including esters, higher alcohols, aldehydes, fatty acids, and terpenoid derivatives [146]. Fruity and floral character are generally associated with ethyl esters (e.g., ethyl hexanoate, ethyl octanoate) and acetate esters (e.g., isoamyl acetate), while higher alcohols contribute to the overall aroma complexity of cider. Aldehydes may be associated with green or oxidative notes, particularly when fruit quality is compromised [125,147].

Recent research highlights that yeast-driven metabolism has a major influence on the aromatic expression of cider, even when the same apple substrate is used. Wei and coworkers [148] showed that fermentations involving *Wickerhamomyces anomalus* alongside *S. cerevisiae* can increase the concentration of key esters and aromatic alcohols, including compounds associated with tropical fruit and floral notes. Although the fermentation strategy is discussed elsewhere in this manuscript, these findings are relevant here because they demonstrate that the aromatic profile of cider from lower-grade apples can be significantly improved, resulting in enhanced sensory complexity.

Yeast strain selection alone can also lead to significant differences in volatile composition. Way and coworkers [149] reported that different yeast strains produced distinct chemical and sensory signatures in apple cider, influencing ester formation, higher alcohol concentrations, and sensory descriptors such as fruitiness and overall balance. Similarly, Wang and coworkers [141] found that yeast strain significantly affected not only aroma-active compounds but also chromatic parameters, confirming that yeast choice influences both volatile expression and visual quality in cider. These studies support the view that the volatile profile of cider is not solely dependent on the apple matrix but is strongly modulated by microbial metabolism.

### 8.3. Phenolics, Mouthfeel, and Astringency

Mouthfeel attributes such as astringency, bitterness, and body are strongly linked to phenolic concentration, degree of polymerization, and interactions with polysaccharides. Phenolic compounds and tannins play a key role in determining bitterness, astringency, and overall mouthfeel in cider matrices, and analytical assessment of these attributes can be influenced by interactions with polysaccharides present in the cider system [150]. Discarded apples may yield juice with altered phenolic extraction patterns due to tissue breakdown, resulting in greater extraction of tannin-like compounds. Increased phenolic levels can enhance complexity and antioxidant potential but may also create sensory imbalance if bitterness and astringency become excessive [132].

Pomace-derived phenolics are particularly relevant because pomace retains a large fraction of apple phenolics even after pressing. Xu and coworkers [31] demonstrated that ciders produced from juices versus corresponding pomaces differed substantially in volatile composition, phenolic-associated sensory perception, and overall sensory attributes, and that cultivar effects interacted with substrate type.

Phenolic composition is also closely related to oxidative stability. Discarded apples may contain oxidized phenolics prior to pressing, which can affect browning intensity and potentially reduce the perception of fresh apple aroma [151]. At the same time, polymerized phenolics may enhance color stability and contribute to a fuller mouthfeel, suggesting that discarded apples could be particularly suited for certain cider styles where deeper color and a structured palate are desirable [152].

### 8.4. Sensory Quality and Consumer Acceptance

Sensory acceptance of cider depends on achieving balance among sweetness, acidity, bitterness/astringency, aroma intensity, and aftertaste. While discarded apples may introduce risks such as oxidative notes or reduced freshness, they can also contribute to complexity, especially when phenolic extraction is elevated. Studies evaluating the effects of yeast strains indicate that sensory descriptors such as fruity aroma, floral character, and overall liking vary widely with fermentation outcomes, demonstrating that cider quality is not inevitably compromised when lower-grade apples are used [141,151].

Aroma enhancement is particularly important for cider from discarded apples, since fruit defects may reduce desirable varietal aroma precursors. Wei et al. reported that cider aroma profiles could be significantly enriched in esters and aromatic alcohols associated with pleasant fruity notes, suggesting that high-quality aromatic profiles can be achieved even in systems with suboptimal raw material quality [148]. This is consistent with the broader concept that cider sensory quality is largely dependent on the final volatile balance rather than on the raw material’s appearance.

Color is another sensory driver influencing consumer perception. Wang and coworkers [141] showed that the yeast strain affected chromatic properties, implying that color management is a pre-fermentation issue and it depends on fermentation-associated chemical transformations [141]. This is especially relevant for discarded apples, which may already show increased browning potential.

## 9. Bioactive Composition, Health Effects, and Upcycling Potential of Cider and Fermented Apple Products

The valorization of discarded, downgraded, or processing-derived apples into cider and related fermented products represents a sustainable strategy aligned with circular-economy principles in the agri-food sector. Beyond reducing food loss, this approach enables the recovery and concentration of bioactive compounds with potential health relevance, while generating value-added products suitable for food, nutraceutical, and non-food applications.

### 9.1. Bioactive Compounds in Apple Pomace and Cider

Apple pomace, the principal by-product of juice and cider production, constitutes a highly enriched matrix of nutritionally and biologically active constituents. It is particularly abundant in polyphenols, including flavonoids, hydroxycinnamic acids, and dihydrochalcones, as well as triterpenes such as ursolic acid, dietary fiber in the form of pectin, and lipids containing health-promoting fatty acids [138,145,153,154,155,156]. These compounds are unevenly distributed within the apple matrix, with peel- and pomace-enriched fractions displaying particularly high concentrations. In such fractions, flavonols and dihydrochalcones may exceed 1 mg/g, while ursolic acid can reach several mg/g, underscoring the significant bioactive potential of apple processing residues [145].

Fermentation processes involved in cider and vinegar production further shape the chemical profile of apple-derived products. Despite biochemical transformations during fermentation, these matrices retain appreciable levels of carbohydrates, organic acids, and antioxidant compounds. Consequently, fermented apple products are promising substrates for the development of functional foods and ingredients with enhanced stability, bioactivity, and sensory attributes [138,157,158]. Table 3 summarizes the main classes of health-relevant bioactives across the apple cider by-product chain.

### 9.2. Health-Related Effects of Cider and Fermented Apple Products

Experimental evidence indicates that the bioactive compounds retained in cider and related products can modulate biological pathways relevant to inflammation and cardiovascular health. In vitro and ex vivo studies have demonstrated that polar lipid fractions isolated from cider and apple juice inhibit human platelet aggregation and platelet-activating factor (PAF)–mediated inflammatory responses. These effects are further supported by favorable omega-6/omega-3 fatty acid ratios, which are commonly associated with cardioprotective potential [144,161].

Fermented apple products, such as vinegar, have also shown a broad spectrum of biological activities. Apple vinegar samples rich in gallic and citric acids exhibited strong antioxidant capacity, as well as anti-inflammatory, antibacterial, and antidepressant-like effects in in vitro assays and animal models [160]. Human intervention data remain limited; however, available studies suggest that moderate apple juice consumption may modestly improve oxidative stress markers and selected cardiovascular risk parameters [162]. Overall, while mechanistic and preclinical findings are promising, clinical evidence supporting health benefits in humans remains preliminary.

### 9.3. Upcycling Strategies and Functional Food Potential

A range of upcycling and valorization strategies has been explored to enhance the functional, nutritional, and economic value of cider by-products. Green extraction technologies, including ultrasound-assisted extraction, enzyme-assisted processes, and supercritical fluid extraction, have been successfully applied to recover polyphenols and triterpenes from apple pomace, yielding extracts with high antioxidant and bioactive potential [145,153,156,159].

Beyond compound recovery, apple pomace has been incorporated into a variety of applications. Its antioxidant and antimicrobial properties support its use in dermal formulations and biodegradable edible films, contributing to both functional performance and sustainability goals [138,154,159]. In beverage reformulation, reintegration of pomace into cider has been shown to enhance phenolic content and antioxidant activity without adversely affecting sensory quality, providing a practical strategy for product fortification [134].

Zero-waste approaches also include converting cider and vinegar pomace into flours, cookies, and other functional food products, thereby effectively closing material loops within the production chain [157,158]. Additionally, the use of scab-damaged or otherwise unmarketable apples for cider production has been shown to be feasible, although changes in the native microbiota and fermentation dynamics require careful control to ensure product quality and safety [128,155].

In summary, discarded apples and cider-processing by-products can be effectively upcycled into cider, vinegar, and functional ingredients while preserving or concentrating polyphenols, triterpenes, pectin, and bioactive lipids. Although experimental and early clinical evidence links these components to antioxidant, anti-inflammatory, anti-platelet, antimicrobial, and potentially cardioprotective effects, most available data come from in vitro or animal studies. Consequently, health-related claims should remain cautious and emphasize moderate consumption, particularly for alcoholic cider, until more robust human intervention studies become available.

### 9.4. Key Differences Between Bioactive Profiles in Cider vs. Apple Juice

Fermentation of apple juice into cider substantially reshapes the quantity and form of bioactive compounds, rather than simply increasing or decreasing them uniformly.

#### 9.4.1. Overall Polyphenol Content

Across multiple cultivars, cider usually contains slightly fewer total phenolics than the corresponding apple juice, but these differences are cultivar-dependent [22,163]. In Finnish cultivars, juices averaged ~48.7 mg/100 mL of total phenolics, while ciders averaged ~37–40 mg/100 mL [163]. In some dessert cultivars, fermentation reduced phenol levels by ~15%, particularly in unripe fruit ciders [26]. However, specific polyphenols (notably free hydroxycinnamates) can increase in cider despite a drop in total content [22,163].

#### 9.4.2. Shifts in Polyphenol Classes

Fermentation alters the phenolic profile of apple products rather than simply changing the total polyphenol concentration. Hydroxycinnamic acids, such as chlorogenic acid and caffeoylquinic acids, are the predominant phenolic compounds in both apple juices and ciders, accounting for approximately 80% of the total phenolic content [22]. During fermentation, the concentration of free hydroxycinnamic acids tends to increase due to the cleavage of glycosidic bonds and the release of bound phenolic forms [22,163].

In contrast, flavonols and dihydrochalcones, including quercetin glycosides and phloridzin, are generally more abundant in apple juices and are significantly reduced in ciders after fermentation [22,163]. Flavan-3-ols and procyanidins, such as catechin, epicatechin, and their oligomers, may be partially lost during fermentation through oxidation, precipitation, or polymerization reactions. However, the extent of these changes can depend on the yeast strain used. For example, fermentations conducted with *S. pombe* have been reported to retain higher levels of procyanidins and (+)-catechin compared with ciders produced using *S. cerevisiae* [22,163].

Fermentation can also lead to the formation or increase in additional metabolites. Nuclear magnetic resonance (NMR) analyses have shown increases in compounds such as tyrosol, trigonelline, chalcones, and several organic acids during fermentation. These compounds contribute to the beverage’s antioxidant profile and may introduce additional health-relevant components characteristic of fermented cider products [164].

#### 9.4.3. Antioxidant Capacity

Apple juice generally has a higher total phenolic content; however, the antioxidant activity of cider remains high after fermentation and is often comparable to that of red wine, while exceeding that of many other beverages [165]. Studies on Spanish and Basque ciders show that their antioxidant capacity, measured using ABTS and FRAP assays, falls within the range typically reported for red wines and is higher than that observed for beverages such as orange juice and beer [165,166]. Although in vitro digestion can reduce the phenolic concentration of cider, a substantial portion of the antioxidant capacity is preserved, with the extent of retention depending on the analytical method used to measure antioxidant activity [166].

#### 9.4.4. Polar Lipids and Antiplatelet Bioactivity

Both apple juice and cider contain polar lipids (mainly phospholipids) rich in monounsaturated fatty acids (MUFA) and n-3 polyunsaturated fatty acids (PUFA), although fermentation can influence their composition and abundance. Apple juices often show higher yields of polar lipids than their corresponding fermented ciders, suggesting that part of the lipid fraction may be metabolized or transformed during fermentation [167]. In terms of lipid classes, phosphatidylcholines tend to dominate in apple juices, whereas phosphatidylethanolamines are more prominent in ciders and appear to be particularly associated with bioactive properties [168]. Across both matrices, lipid profiles generally display favourable n-6/n-3 fatty acid ratios below 5. In several cider samples, the ratios are even lower, typically between 0.5 and 2.5, which supports a stronger potential for anti-inflammatory and antiplatelet biological activity [168].

#### 9.4.5. Influence of Variety, Ripeness, and Processing

The apple cultivar is one of the strongest determinants of phenolic concentration in both juice and cider. Traditional cider apple varieties can reach polyphenol levels of approximately 261–970 mg/L in juice, whereas juices produced from dessert apples generally contain lower concentrations, around 154–178 mg/L [169,170]. The stage of fruit ripeness also affects the initial composition of apple juice and can influence phenolic changes during fermentation. Unripe apples typically contain higher phenolic concentrations, although they may experience greater phenolic losses during fermentation [22,26]. In addition, the yeast species and strain used for fermentation, such as *S. cerevisiae* or *S. pombe*, as well as different commercial strains, can significantly affect the release of hydroxycinnamic acids and the retention of procyanidins, thereby shaping the final phenolic composition of cider [22,163]. Table 4 shows the contrasting major bioactive classes between juice and cider.

#### 9.4.6. Health-Related Implications

Apple juice typically contains higher total polyphenol concentrations, particularly flavanols and dihydrochalcones, which may confer stronger direct antioxidant capacity per unit volume. In contrast, cider retains a substantial proportion of apple phenolics—especially hydroxycinnamic acids and certain procyanidins—while also developing additional compounds during fermentation. These include fermentation-derived metabolites and bioactive polar lipids that have been associated with anti-platelet and anti-inflammatory activity [165,166,167,168,169]. However, the presence of ethanol in cider limits its potential health benefits despite the presence of these bioactive components.

## 10. Emerging Cider Styles and Product Innovation

Alcohol consumption in many European countries has declined steadily in recent decades [169]. In contrast, cider sales have continued to grow, increasing the beverage’s presence in the international market [170]. The development of new and diverse cider flavors may further attract consumers and expand market share [147].

Cider, one of the oldest fermented beverages produced from apple juice, has undergone significant transformation in recent decades. Traditionally associated with regional production systems, the sector is now experiencing renewed global interest driven by technological advances, changing consumer preferences, and the expansion of the craft beverage industry. This renewed attention has stimulated innovation in raw materials, fermentation technologies, product styles, and marketing strategies [32,171].

### 10.1. Raw Materials and Apple Cultivar Selection

The composition of apples used in cider production plays a fundamental role in determining the chemical and sensory characteristics of the final beverage. Apples contain fermentable sugars, organic acids, phenolic compounds, and aroma precursors that directly influence fermentation behavior and flavor development [6,33]. Consequently, the selection of apple cultivars is a key factor in cider quality and style differentiation.

Recent studies demonstrate that different apple varieties produce significant differences in alcohol content, acidity, amino acid composition, and volatile aromatic compounds in cider [31,61]. For instance, research comparing several cultivars, including Fuji, Golden Delicious, and Granny Smith, showed that cultivar choice strongly affects both physicochemical properties and aroma profiles, highlighting the importance of raw material selection in cider production. For instance, Fuji apples produced the highest alcohol levels, followed by Golden Delicious, while Granny Smith resulted in the lowest alcohol content. Differences among cultivars also affected the profiles of organic acids, amino acids, and volatile compounds. Multivariate analyses, including principal component analysis and cluster heat mapping, classified the ciders into three distinct groups, indicating that although the samples shared some nutritional and flavor characteristics, each group exhibited unique compositional and sensory features [172].

The sugar composition of apples is particularly important because it determines potential alcohol yield during fermentation. Apple fruit typically contains a mixture of fructose, glucose, and sucrose, with fructose generally being the dominant sugar [32]. Genetic factors, environmental conditions, and maturity stage influence the balance between these sugars, which in turn affects yeast metabolism and fermentation performance. In addition, modern breeding programs are increasingly focused on developing apple cultivars specifically adapted for cider production, with the aim of enhancing desirable attributes such as sugar concentration, phenolic composition, and fermentation efficiency [33].

### 10.2. Flavor and Sensory Characterization

The sensory profile of cider is largely determined by its volatile and semi-volatile compounds, including esters, alcohols, aldehydes, and organic acids, which originate from both the raw materials and the fermentation process. Among these compounds, esters are particularly important because they impart fruity and floral aromas characteristic of many cider styles. Riper apples tend to contain higher levels of esters and higher alcohols, contributing to a more complex aromatic profile. As a result, cider producers often use very ripe or even senescent fruits to enhance aroma intensity [26].

Apples contain over 300 volatile aromatic compounds, though only a fraction of these are transferred to the final cider [173]. Among the most abundant esters in cider are isoamyl acetate, hexyl acetate, and ethyl octanoate, while compounds such as 2-phenylethanol and terpenes, though present in lower concentrations, contribute positively to aromatic complexity [173,174].

Recent analytical studies of commercial ciders have identified numerous volatile and semi-volatile compounds that shape aroma complexity and differentiate products. Multivariate statistical analyses, such as principal component analysis and cluster heat mapping, have been used to link chemical composition with sensory attributes and classify cider styles based on their volatile profiles. These approaches provide valuable tools for understanding how production methods influence flavor development and support innovation in cider production [175].

The growth of the global cider market has been closely linked to shifting consumer preferences and rising demand for craft and artisanal beverages. Studies suggest that cider consumers can be divided into distinct segments based on preferences for flavor attributes or production information [32]. Some consumers prioritize sensory characteristics, such as sweetness, fruitiness, and aromatic intensity, while others focus on production methods, geographical origin, authenticity, or sustainability. These trends have encouraged producers to develop new cider styles and product formats, including fruit-infused, barrel-aged, and low-alcohol ciders, enabling differentiation in competitive markets [146,176].

## 11. Valorization of Cider By-Products: Pomace and Beyond

Apple pomace is the solid residue left after apples are pressed during juice extraction and is a by-product of the process. It consists mainly of seeds, peel, and pulp and can account for up to 30% of the original fruit mass, depending on the cultivar and processing method [177]. Global apple processing is estimated to produce roughly 6–12 million tonnes of pomace each year, and much of it is still sent to landfill despite its valuable components, such as pectin, phenolic compounds, and dietary fibers [178]. Apple pomace can be used as cattle feed, as orchard fertilizer, or dried for pectin production. Because pomace is rich in dietary fiber and polyphenols and shows substantial in vitro antioxidant activity, there is growing interest in using it for human nutrition as well as for pharmaceutical and cosmetic applications [93]. Apple pomace is increasingly used to extract value-added products such as dietary fiber, natural antioxidants, and biopolymers. Because of their strong free-radical-scavenging properties, the antioxidants extracted from apple pomace have potential applications as food supplements and nutraceuticals. Additionally, the high carbohydrate content of apple pomace makes it a substrate for various microbial processes that produce organic acids, enzymes, and ethanol [179].

### 11.1. Upcycling Cider Pomace for Food Applications

Pomace is a great source of fiber and carbohydrates, but is low in protein and can be fed directly to dairy cows and store cattle. It must be fresh, not moldy or acetified, and if it is high in phenolic, it should be added to the diet gradually to prevent scouring [180]. Due to its low pH and high carbohydrate content, apple pomace also ensiles readily.

Apple pomace contains valuable macro- and micronutrients, including sugars, lipids, phenolic bioactive compounds, minerals, and dietary fiber. Apple pomace contains a large number of polyphenols, which can reduce oxidative stress. There is a strong correlation between the phenolic content of apples and the antioxidant activity of this fruit [181]. However, the overall phenolic content in apples is highly dependent on apple variety, ranging from 200 to 8000 mg GAE/100 g (expressed in gallic acid equivalents, GAE) [144]. Environmental factors, including growing conditions, weather effects, annual variations, varietal characteristics, fruit age, post-harvest treatments, processing techniques, and storage conditions, can all contribute to these variations. Apple pomace is also a source of bioactive lipid compounds [182]. Likewise, the lipid content in apple pomace varies depending on the apple variety from which it is derived. Apple pomace is a natural source of pectic substances, containing approximately 10–15% pectin on a dry-weight basis. In pomace, pectin occurs mainly as protopectin, an acid-soluble polysaccharide [183].

Given its rich fiber content, including pectin and starch, apple pomace can be incorporated into a variety of food products, such as jellies, jams, and other edibles. Additionally, due to its many health benefits, apple pomace is increasingly being used in a variety of bio-functional foods, supplements, and nutraceuticals [144].

Apple pomace can be a source of dietary fiber in bakery product formulations and of polyphenols, thereby increasing the antioxidant properties of these foods [183]. Because apple pomace has a pleasant fruity flavor and brownish color, it can also improve the sensory properties of bakery products. The partial replacement (15%) of wheat flour by apple pomace in the formulation of cookies was associated with a positive effect on the total phenolic content, antioxidant capacity, and the concentration of phenolic acids and flavonoids in cookies, therefore, enhancing the quality of cookies with respect to the presence of phenolic compounds and antioxidant capacity [184]. A previous study [185] showed that biscuits containing 15% of apple pomace presented better physicochemical, technological, and sensory properties. Similarly, adding 10% apple pomace powder to wheat flour to produce apple pomace-enriched bread resulted in a bakery product with high nutritional value and adequate quality and sensory properties [186].

In the meat production industry, apple pomace can be used to increase dietary fiber. Freeze-dried apple pomace was incorporated in minced beef meat at 4% and 8% levels to obtain fortified burgers [187]. The enrichment of red meat with apple pomace resulted in hygiene and safety characteristics comparable to those of non-fortified products, without causing alterations such as rapid spoilage or a consequent reduction in shelf life. However, the sensory analysis results showed that beef burgers fortified with 8% apple pomace had lower scores for elasticity, cohesiveness, juiciness, and fattiness than those with 4% and 0%, which were very similar. Another study successfully developed turkey sausages by incorporating freeze-dried apple pomace at 3%, 5%, and 8% levels in turkey breast meat, finding that a 3% level produced sausages with physicochemical and sensory properties comparable to the control, while enhancing antioxidant activity and fiber content [188]. However, higher levels of freeze-dried apple pomace resulted in declines in sensory attributes, including texture and overall acceptability. In a study conducted by Kęska and coworkers [189], enriching ground pork with freeze-dried apple pomace (0.5% or 1%) yielded a high-quality product comparable to unenriched products, without causing rapid deterioration or shortening shelf life. However, a slight increase in bacterial content, including coliforms, was observed in variants with apple pomace.

In a study using freeze-dried apple pomace as a natural stabilizer and texturizer in set-type yogurt, the addition of apple pomace (0.1%, 0.5% and 1% *w*/*w*) before fermentation favored aggregation of casein micelles at an early stage of fermentation causing onset of gelation at higher pH compared to the control, shortened the fermentation time, and eventually developed a firmer and more consistent yogurt gel during cold storage, especially the yogurt fortified with 0.5% apple pomace [190]. In another study [191], the addition of apple pomace powder (0.2–1.0%) reduced yogurt fermentation time by 1 h and significantly increased antioxidant activity, which correlated with apple pomace content. The addition of apple pomace significantly affected the textural properties of the yogurt during storage (20 days), with firmness values ranging from 1390.3 g (control sample) to 2244.5 g (1% apple pomace). Likewise, Klojdova and coworkers [192] found that apple pomace powder at concentrations of 1, 2, and 4%, obtained by freeze-drying or air-drying, particularly at higher concentrations, improved the hardness and elasticity of yogurt gels.

### 11.2. Upcycling Cider Pomace for Non-Food Applications

Chemicals and biofuels can be produced sustainably from apple pomace. It can be used to produce organic acids, antioxidants, and enzymes, and fermentation processes can convert it into bioethanol and biobutanol.

Following a biorefinery approach, Molinuevo-Salces and coworkers [193] successfully produced bioethanol and methane from apple pomace. Different strains of the yeasts *Saccharomyces cerevisiae*, *Kluyveromyces marxianus*, *K. lactis*, and *Lachancea thermotolerans* were used for bioethanol production. In general, all *Kluyveromyces* and *Lachancea* strains obtained bioethanol concentrations between 49.9 and 51.5 g/L. The performance of *S. cerevisiae* strains was more variable, with ethanol values ranging from 25.7 to 51.0 g/L. In the same study, high methane yields were obtained during anaerobic co-digestion of swine manure at low substrate concentration and apple pomace at low content in the mixtures. Although the biorefinery design is the most eco-efficient option for co-producing bioethanol and biogas, economic barriers and the low sugar content of apple pomace remain key challenges to implementation [194].

Butanol is an excellent biofuel alternative to ethanol and can be produced via acetone-butanol-ethanol (ABE) fermentation of apple pomace sugars using bacteria of the genus *Clostridium*. In a study on acid crash of ABE fermentation [195], an undesirable phenomenon characterized by a total arrest of cell growth and solvent production, apple pomace hydrolyzate was used as a substrate for producing butanol. Operational parameters that may influence the prevention of acid crash occurrence, such as pH, CaCO_3_ concentration, and culture temperature, were optimized in *Clostridium beijerinckii* CECT 508 cultures. This study demonstrated that higher pH and CaCO_3_ concentration, and lower temperatures prevent acid crash and favor butanol production. Optimal conditions were pH 7, 6.8 g/L CaCO_3_, and 30 °C. In these conditions, 107.33 g of butanol, 65.56 g of acetone, and 2.69 g of ethanol were obtained per kg of dry apple pomace. In another study, by Jin and coworkers [196], on ABE fermentation of soluble and hydrolyzed sugars in apple pomace by *Clostridium beijerinckii* P260, a total of 202.8, 42.1, 41.4, 260.1, and 262.2 g of overall ABE (acetone + butanol + ethanol) was produced from each kg of dry apple pomace using water soluble sugars (WSS), acid hydrolyzed sugars (ACHS), alkali hydrolyzed sugars (ALHS), WSS + ACHS, and WSS + ALHS as the fermentation substrates, respectively.

Biodegradable waste is frequently managed through composting. Apple pomace can be successfully used as a composting substrate. However, apple pomace is acidic, has high moisture content, and low nitrogen content. Consequently, it should be used in conjunction with other waste types to produce compost [197]. Vermicomposting results from the ingestion, digestion, and absorption of organic waste carried out by earthworms. Hanc and Chadimova [198] showed that vermicomposting, using earthworms of the genus Eisenia, is a suitable technology for the decomposition of apple pomace waste into a value-added product. The resulting vermicomposts were rich in available K (9899–15,878 mg/kg), and the available P content was approximately one order of magnitude lower (1018–1779 mg/kg), while the available Mg content ranged from 435 to 787 mg/kg.

## 12. Conclusions

The valorization of discarded apples through cider production represents a robust and sustainable strategy to mitigate food waste while generating value-added products within a circular economy framework. Despite their exclusion from fresh markets, discarded apples generally retain adequate levels of fermentable sugars, organic acids, phenolic compounds, and micronutrients required for successful fermentation. However, their inherent variability, arising from differences in cultivar, maturity, storage conditions, and degree of damage, introduces significant challenges for process standardization and product consistency.

This review demonstrates that the interaction between raw material composition, nutrient availability, and yeast metabolism primarily governs fermentation performance and cider quality. In particular, the frequent limitation and variability of YAN, together with micronutrient imbalances, constitute critical constraints in cider fermentation from discarded apples. Consequently, targeted nutrient management strategies, combined with appropriate yeast selection, are essential to ensure fermentation efficiency, prevent off-flavor formation, and achieve consistent sensory outcomes.

Yeast strategy has evolved toward a modular approach, in which *Saccharomyces* strains provide reliability and fermentation completeness, while non-*Saccharomyces* yeasts and mixed fermentations enable the modulation of aroma complexity, acidity, and mouthfeel. In parallel, fermentation induces significant biochemical transformations, shifting the phenolic profile of apple substrates toward increased availability of free hydroxycinnamic acids and fermentation-derived metabolites, while generally preserving relevant antioxidant capacity despite partial losses in total phenolics.

Advances in fermentation technologies, including controlled inoculation strategies, immobilized-cell systems, and innovative pretreatment methods, further enhance process efficiency and adaptability to variable raw materials. Moreover, integrating cider production into broader upcycling strategies enables the valorization of by-products such as apple pomace into functional ingredients rich in polyphenols, dietary fiber, triterpenes, and bioactive lipids.

Discarded apples should be regarded not as inferior raw materials but as heterogeneous substrates requiring informed technological intervention. When properly managed, they can yield ciders with acceptable or even enhanced sensory complexity. At the same time, cider and its derived products represent promising carriers of bioactive compounds with reported antioxidant, anti-inflammatory, and cardioprotective potential. However, as current evidence is largely based on in vitro and preclinical studies, health-related claims should be approached with caution and aligned with recommendations for moderate consumption, particularly in the case of alcoholic beverages.

Future research should focus on improving the predictability of fermentation under variable raw-material conditions, optimizing integrated nutrient and yeast management strategies, and strengthening clinical evidence of the health effects of cider-derived bioactive compounds. Such efforts will be essential to support the scalable and scientifically grounded valorization of discarded apples in the cider industry.

## Figures and Tables

**Table 1:** Typical defect types in discarded apples and their potential technological implications for cider production.

Defect Type	Primary Physiological/Biochemical Effect	Potential Fermentation Consequence	Possible Mitigation Strategy	Ref.
Bruising/Mechanical damage	Polyphenol oxidase activation; phenolic oxidation; tissue disruption; increased susceptibility to fungal contamination	Increased browning; modified phenolic extraction; possible changes in astringency and color; elevated microbial load	Rapid processing; trimming; antioxidant use; clarification; sanitation control	[6,14,17]
Internal browning	Oxidative stress; membrane degradation; enzymatic browning reactions	Altered phenolic profile; potential reduction in fresh apple aroma perception	Sorting, blending with sound fruit; controlled oxidation management	[15,16,21]
Superficial cosmetic defects (size, peel blemishes, color heterogeneity)	Generally, minimal impact on intrinsic sugar, acid, or nitrogen composition	Negligible direct fermentation impact; largely aesthetic rejection	Direct processing; standard quality control	[19]
Prolonged storage/Senescence	Changes in sugar–acid balance; altered nitrogen availability; increased oxidative reactions	Fermentation variability, potential nutrient limitation, and altered acid balance	Acid adjustment; yeast nutrient supplementation; blending strategies	[6,20]
Fungal infection/Microbial deterioration	Increased microbial diversity; possible mycotoxin (e.g., patulin) risk; competition with inoculated yeast	Fermentation instability; off-flavor formation; safety concerns	Rigorous sorting; washing; trimming; mycotoxin testing; controlled inoculation	[6,17]

**Table 3:** Main classes of health-relevant bioactives across the apple cider by-product chain.

Compound/Group	Main Source in the Chain	Reported Bioactivity/Role	Ref.
Polyphenols (quercetin, dihydrochalcones, catechin, taxifolin, eriodictyol)	Pomace, peels, fermented pomace	Antioxidant, anti-inflammatory, antimicrobial, cardioprotective potential	[145,154,155,156]
Triterpenes (ursolic acid)	Peel/pomace	Linked to antioxidant and metabolic benefits	[143,154]
Pectin/dietary fibre	Pomace	Prebiotic effects, texturizing/film-forming agent	[138,153,159]
Polar lipids (PC, PE rich in MUFA/PUFA)	Cider, by-products	Antiplatelet, anti-inflammatory, cardioprotective	[149]
Organic/acetic acids	Vinegar from apples	Antimicrobial activity; contributes to metabolic effects	[155,160]

**Table 4:** Contrasting major bioactive classes between apple juice and cider.

Feature/Class	Apple Juice (Pre-Fermentation)	Cider (Post-Fermentation)	Ref.
Total phenolics	Generally higher	Slightly lower overall; cultivar-dependent	[22,26,163]
Hydroxycinnamic acids	Mostly conjugated forms	Higher proportion of free hydroxycinnamates	[22,163]
Flavonols & dihydrochalcones	Higher concentrations	Markedly reduced	[22,163]
Procyanidins/flavan-3-ols	High; partly sensitive to oxidation	Lower or redistributed; retention depends on yeast/cultivar	[22,30,163]
Fermentation-specific phenolics	Absent	Tyrosol, chalcones, and other metabolites increased	[164]
Polar lipids (PC, PE)	Higher PL yield; PC is the most bioactive	Lower PL yield, more PE; often improved n-6/n-3 ratio	[168]
Antioxidant capacity	High (variety-dependent)	Remains high; similar to red wine in many ciders	[163,164]

PL—Polar lipids; PC—Phosphatidylcholine; PE—Phosphatidylethanolamine.

## Data Availability

No new data were created or analyzed in this study. Data sharing is not applicable to this article.
